# Supplementary material for: Unique spicules may confound species differentiation: taxonomy and biogeography of Melonanchora Carter, 1874 and two new related genera (Myxillidae: Poecilosclerida) from the Okhotsk Sea
Source: PeerJ. 2021 Dec 22;9:e12515. doi: 10.7717/peerj.12515 (PMC8710052; doi:10.7717/peerj.12515)
Supplement: Supplemental Information 1 [file peerj-09-12515-s001.docx]

**Key to *Melonanchora***

| 1. With styles as choanosomal megascleres | 2 |
| --- | --- |
| 2. With strongyles as choanosomal megascleres | 4 |
|  |  |
| 2. Chelae with reduced alae | *Melonanchora maeli* |
| 3. Chelae without reduced alae | 3 |
| 3. With elliptical spherancorae and chelae with protruding alae | *Melolonanchora elliptica* |
| 4. With somewhat irregularly oval spherancorae and chelae with straight alae | *Melonanchora insulsa* |
| 4. With two clearly differentiated megascleres categories between the ectosome and the choanosome | *Melonanchora tumultuosa* |
| 5. With just a single megasclere category | 5 |
| 5. Spherancorae smaller or equal in size than the biggest chelae category | *Melonanchora emphysema* |
| 6. With a third category of chelae, with reduced teeth and slightly unequal ends | *Melonanchora intermedia* |
